# Quantification of Carnosine and Anserine in Diverse Meat Matrices via Optimized Extraction and Validated HPLC-DAD

**DOI:** 10.3390/mps9050131

**Published:** 2026-09-04

**Authors:** Chalita Kaewkhow, Chokwan Chumang, Piboon Pantu, Patraporn Luksirikul, Prapasiri Pongprayoon, Skorn Koonawootrittriron, Thanathip Suwanasopee, Tharinee Saleepoch, Sutasinee Kityakarn

**Affiliations:** 1 Department of Chemistry, Faculty of Science, Kasetsart University, Bangkok 10900, Thailand; chalita.kaewk@ku.th (C.K.); chokwan.c@outlook.com (C.C.); fscipbp@ku.ac.th (P.P.); fscipplu@ku.ac.th (P.L.); fsciprpo@ku.ac.th (P.P.); 2 Center for Advanced Studies in Nanotechnology for Chemical, Food and Agricultural Industries, KU Institute for Advanced Studies, Kasetsart University, Bangkok 10900, Thailand; 3 Advanced Porous Materials for One Health Integrations (APM Unit), Department of Chemistry, Faculty of Science, Kasetsart University, Bangkok 10900, Thailand; 4 Department of Animal Science, Faculty of Agriculture, Kasetsart University, Bangkok 10900, Thailand; agrskk@ku.ac.th (S.K.); agrtts@ku.ac.th (T.S.)

**Keywords:** carnosine, anserine, HPLC-DAD, Thai native chicken, functional food

## Abstract

Carnosine and anserine are bioactive, histidine-containing dipeptides recognized for their antioxidant, pH-buffering, and neuroprotective functions. However, their precise quantification is frequently compromised by their high structural similarity and complex matrix-induced analytical interferences. This study developed and validated an optimized, simplified extraction protocol coupled with high-performance liquid chromatography diode array detection (HPLC-DAD) for the simultaneous determination of both dipeptides across diverse meat matrices, including Thai native black-bone chicken (Nin-Kaset), commercial broiler, pork, beef, and buffalo meat. Extraction efficiency was systematically optimized by modulating the solution pH. Under optimized conditions, the validated method demonstrated excellent linearity (1–200 µg mL^−1^, r^2^ ≥ 0.9990) with limits of detection and quantification (LOD/LOQ) values at 0.0093/0.0283 mg g^−1^ for carnosine and 0.0139/0.0420 mg g^−1^ for anserine. Method accuracy was confirmed by satisfactory recoveries ranging from 96.64% to 102.68%, with repeatability values (RSDs) of 0.70–14.18%. Application to real matrices revealed distinct dipeptide profiles. Pork contained the highest carnosine level (3.59 ± 0.19 mg g^−1^), whereas white-feather Nin-Kaset exhibited the highest anserine level (6.77 ± 0.40 mg g^−1^). Overall, the validated framework offers a rapid, cost-effective, and robust approach for routine dipeptide profiling, supporting future applications in food authentication, nutritional evaluation and functional food development.

## 1. Introduction

The global landscape of meat consumption has undergone a dramatic expansion over the past few decades, driven by population growth, urbanization, and shifting dietary preferences. Notably, poultry production and consumption have grown far more rapidly than red meat alternatives, largely due to its desirable nutritional profile characterized by high crude protein and low intramuscular fat contents. Between 1990 and 2023, global consumption of pork, beef, sheep, and chicken increased, especially chicken [1]. In 1990, chicken meat made up 19.21% of global meat consumption, ranking below pork and beef. However, it had risen to 39.55% by 2023, making it the most consumed type of meat globally. Global chicken production had risen to 27.10% from 2012 to 2025 [2]. The USDA reported that protein and total fat contents in chicken are 22.5 and 1.93 g/100 g, which are less than pork (21.6 g/100 g for protein and 3.90 g/100 g for total fat) and beef (21.1 g/100 g for protein and 6.46 g/100 g for total fat).

Concurrently, the increasing awareness of diet and health relationships has driven people to seek functional foods that not only provide basic nutrition but are also enriched with bioactive compounds. Bioactive compounds are naturally occurring food constituents that contain beneficial health properties [3]. These compounds include phenolics [4,5], carotenoids [4,6], prebiotics [7,8], and probiotics [7,9]. Antioxidant activity is one of the therapeutic properties of bioactive compounds. Chicken meat also contains histidine-containing dipeptides such as carnosine (β-alanyl-L-histidine) and anserine (β-alanyl-3-methyl-L-histidine), which are well known for their antioxidant function [10,11,12].

Carnosine and anserine are endogenous dipeptides dominantly found in skeletal muscle and the nervous system of vertebrates. Both compounds show physiological and biological activities, for example, antioxidant [13,14], anti-inflammatory [13], immunomodulatory [13], antiaging [13], antidiabetic [13,14], anticancer [15], cognitive function [13,16,17,18,19], and pH buffering [13]. Moreover, carnosine has been studied for its neuroprotective effects against Alzheimer’s disease. Carnosine shows significant potential for inhibiting the accumulation of β-amyloid protein in the brain that causes this disease [11,16] and is also a precursor of GABA, the inhibitory neurotransmitter in the brain, for neuroprotective action by chelating Zn^2+^ and Cu^2+^, which is related to neurotoxicity [11,20]. In addition, recent studies reported that concentrations of carnosine and anserine vary significantly among animal species. Red meat, such as pork and beef, contains higher levels of carnosine, whereas poultry meat, especially chicken meat, contains higher levels of anserine [11,21,22].

Native chicken is considered a valuable source of functional food because it contains higher levels of bioactive compounds compared with broiler, including creatine, carnitine, carnosine, and anserine [12,23,24,25]. Nin-Kaset, a Thai native black-boned chicken, was developed by the Tropical Animal Genetic Special Research Unit (TAGU) from Kasetsart University. The chicken was bred between Mongolian black-boned chicken and Khek Noi black-boned chicken from Phetchabun province, aiming to improve meat quality and conserve the Thai native black-boned breeds. Nin-Kaset has distinctive properties such as a unique feathered chicken comb and three feather colors (white, black, and red-brown with freckles). Therefore, native chicken has been recognized as a functional food with beneficial nutrition for consumers.

Although the therapeutic properties of carnosine and anserine have been widely researched, it is difficult to quantify the exact amount of both compounds in meat. Their polarity and structural similarities often interfere with chromatographic separation, resulting in low resolution for quantification. A previous study reported the incomplete separation of carnosine and anserine due to similar polarities of both analytes [26,27]. It is important to identify and quantify the exact amounts of carnosine and anserine in meat. In addition, several analytical techniques have been used to determine carnosine and anserine, including high-performance liquid chromatography (HPLC) [11], liquid chromatography–tandem mass spectrometry (LC–MS) [10,28], liquid chromatography–tandem mass spectrometry (LC–MS/MS) [27,29], pure micellar liquid chromatography [22], capillary electrophoresis (CE) [30], and voltammetry [31]. However, the limitations of these techniques are expensive instruments, an expert analyst for operation, and long-time analysis. Among these available techniques, HPLC is a widely used quantification technique for the determination of carnosine and anserine in meat samples [10,11,21,22]. HPLC provides accurate and precise resolution without adding derivatives or additives. The determination of carnosine and anserine based on the separation of charged dipeptides due to the ion exchange HPLC [32,33] and ion-pair HPLC [34] were used. Nevertheless, ion-pair HPLC requires the addition of an ion-pair reagent to the mobile phase, which may cause prolonged column equilibration and mobile phase preparation. In contrast, ion exchange can be separated through ionic interactions with the stationary phase without requiring additional reagent in the mobile phase. Therefore, ion exchange HPLC was employed in this study to determine carnosine and anserine.

To address current gaps, this study aimed to develop and validate a simplified, rapid, low-cost and effective sample preparation protocol coupled with an underivatized HPLC-DAD method operating at ambient temperature for the simultaneous determination of carnosine and anserine. The extraction efficiency was systematically optimized under mild conditions by adjusting solution pH and acidic solvent-to-sample ratios to minimize matrix interferences. The validated workflow was successfully applied to map the distinct dipeptide profiles across a diverse suite of commercial and native meat matrices, including pork, beef, buffalo meat, broiler, and the indigenous Nin-Kaset black-boned chicken. The findings underscore the role of Nin-Kaset as an exceptionally rich source of anserine, validating its position as a high-value functional food for health-conscious consumers.

## 2. Materials and Methods

### 2.1. Sample Preparation

Five meat cut samples of broiler breast, pork, and beef were purchased from three different commercial brands in a supermarket in Bangkok, Thailand. The white-feather Thai native chicken breast, black-feather Thai native chicken breast, and buffalo meat were supplied from the Tropical Animal Genetic Special Research Unit (TAGU) from Kasetsart University, Bangkok, Thailand. The total number of cut meats was 65 samples for five quantitative replications each. These were immediately frozen at −20 °C and minced before the extraction process.

### 2.2. Standard Solution Preparation

Stock standard solutions of anserine (C10H16N4O3, Toronto Research Chemicals, Vaughan, ON, Canada) and L–carnosine (C9H14N4O3, Glentham Life Sciences, Wiltshire, UK) at a concentration of 1000 µg mL^−1^ were prepared in sodium dihydrogen phosphate buffer (NaH2PO4, M&B, Birmingham, UK) adjusted to pH 5.0 by 0.01 M potassium hydroxide solution (KOH, QRëC, Auckland, New Zealand). The working standard solutions were prepared by diluting in acetonitrile (CH3CN, Samchun, Seoul, Republic of Korea) and 50 mM adjusted phosphate solution (3:1, *v*/*v*). The prepared stock standard solutions were used to create a calibration curve in the concentration range 1–200 µg mL^−1^.

### 2.3. Extraction Method

Each minced meat sample (1.0 g) was added to 3 mL of 0.01 M hydrochloric acid (37% HCl, QRëC, New Zealand). The mixture solution was vortexed and sonicated for 5 min. Then the mixture was centrifuged at 4 °C and 10,000 rpm for 20 min; 1 mL of the supernatant was transferred into a 5 mL volumetric flask and adjusted to volume with a mixture solution of acetonitrile and adjusted phosphate solution (3:1, *v*/*v*). After that, 1.00 mL of the solution was mixed with 3.00 mL of acetonitrile as a protein precipitation solution and left at room temperature for 20 min. Next, the mixture was centrifuged at 4 °C at 10,000 rpm for 10 min. The supernatant was filtered through a syringe filter (0.22 µm PTFE-H_2_O) into a 1.5 mL HPLC-vial.

### 2.4. High-Performance Liquid Chromatography Analysis (HPLC)

Chromatographic separation was performed on an ion exchange column (Asahipak NH2P–50 4E, 4.6 × 250 mm, 5 µm, Tokyo, Japan) with a guard column of the same material using the Shimadzu (Kyoto, Japan) LC–20A HPLC system equipped with a photodiode array UV detector at ambient temperature. The elution was conducted using a 45:55 (*v*/*v*) ratio of CH_3_CN and 50 mM NaH_2_PO_4_ with a flow rate of 1.0 mL/min. The injection volume was 60 μL. The UV detection was conducted at 210 nm. The analysis time was completed within 13 min.

### 2.5. Method Validation Procedure

The analytical method was validated in accordance with AOAC guidelines, evaluating linearity, sensitivity, accuracy, and precision.

#### 2.5.1. Linearity and Range

Calibration curves for carnosine and anserine were constructed over the concentration range of 1–200 µg mL^−1^ using five calibration levels. Linearity was considered acceptable when the coefficient of determination (r^2^) exceeded 0.999. The limits of detection (LOD) and limits of quantification (LOQ) were estimated based on the signal-to-noise (S/N) approach derived from the calibration data for both analytes.

#### 2.5.2. Accuracy

Method accuracy was assessed through spike–recovery experiments at three nominal spiking levels (10, 50, and 200 µg mL^−1^), with five replicates at each level. Recovery percentages were calculated by comparing the measured concentrations with the theoretical spiked amounts. Acceptable recoveries were defined within the range of 80–120%.

#### 2.5.3. Precision

Repeatability (intra-day precision) was evaluated by analyzing carnosine and anserine standards at three concentration levels (1, 50, and 200 µg mL^−1^), each in five replicates. Method precision was expressed as the relative standard deviation (RSD, %) calculated for each concentration level.

### 2.6. Statistical Analysis

The data were analyzed by two-way analysis of variance (ANOVA) using the SAS OnDemand for Academics version 9.3M2 (SAS Institute Inc., Cary, NC, USA), with meat type and suppliers as the two independent factors. All data were expressed as the means ± standard deviations (SD). Significant effects were performed by Duncan’s multiple-range test at the level of *p* < 0.05.

## 3. Results and Discussion

### 3.1. Optimization of the Simplified Extraction Protocol

**Figure 1:**
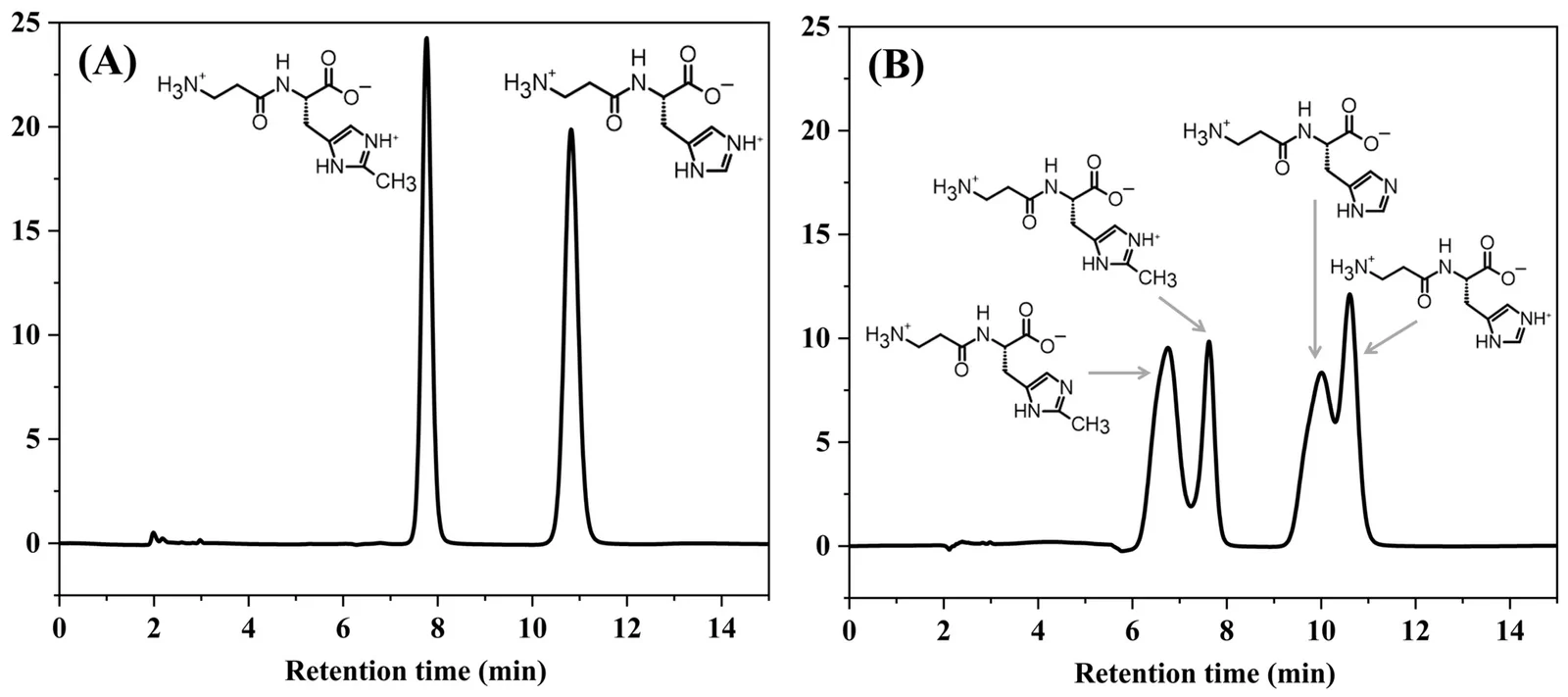
HPLC chromatogram to study the effect of pH 300 µg mL^−1^ mix standard of carnosine and anserine in (**A**) CH_3_CN and adjusted phosphate solution (3:1) and (**B**) ultrapure water. Both chromatograms performed under the 45:55 (*v*/*v*) ratio of CH_3_CN and 50 mM NaH_2_PO_4_ elution at 210 nm.

Carnosine (H_3_Car) and its methylated derivative, anserine (H_3_Ans), possess three distinct ionizable functional groups: an imidazole ring, an amino group, and a carboxylic acid group. Consequently, their protonation states, overall molecular polarity, and subsequent chromatographic behaviors are highly dependent on the pH [31]. To systematically evaluate the influence of pH on acid-base speciation and HPLC performance, a 300 µg mL^−1^ mixed standard of carnosine and anserine was prepared in two different diluents: an acetonitrile-adjusted phosphate solution mixture (CH_3_CN:adjusted NaH_2_PO_4_ solution, 3:1, *v*/*v*, Figure 1A) and ultrapure water (Figure 1B). The amounts of anserine and carnosine were determined by high-performance liquid chromatography (HPLC) using a 45:55 (*v*/*v*) ratio of CH_3_CN and 50 mM NaH_2_PO_4_ elution at a wavelength of 210 nm.

As shown in Figure 1A, the standard solution diluted in a CH_3_CN:adjusted NaH_2_PO_4_ solution (3:1 *v*/*v*) measured at an equilibrium pH of 5.20. At this pH, both anserine and carnosine predominantly existed as single, positively charged protonated species (H_2_Ans^+^ and H_2_Car^+^). This uniform ionization state yielded well-defined, systematic single peaks with stable retention times of 7.73 min for anserine and 10.83 min for carnosine.

Conversely, dilution of the standard in ultrapure water resulted in a shifted equilibrium pH of 6.06, which is close to the reported second dissociation constant (pK_a2_) values of anserine (7.04) and carnosine (6.79); both analytes underwent partial deprotonation. Operating near these pK_a2_ values suppressed the zwitterionic character of the dipeptides and promoted chemical speciation heterogeneity. As a result, multiple ionic forms coexisted in solutions, leading to inconsistent interactions with the stationary phase. This equilibrium flux manifested chromatographically as distinct doublet peaks for both analytes (anserine: 6.34 and 7.66 min; carnosine: 9.55 and 10.68 min). Similar to the standard in adjusted phosphate solution, the doublet peaks of carnosine and anserine were shown in Figure S1.

These findings highlight that failing to strictly control the pH near the analytes pK_a_ values compromises chromatographic resolution and peak symmetrical peak [31,35]. To ensure method robustness, reproducibility, and quantitative reliability when analyzing complex, diverse meat matrices, stringent pH control is mandatory during both sample extraction and mobile phase preparation.

### 3.2. Method Validation

#### 3.2.1. Linearity and Range

**Figure 2:**
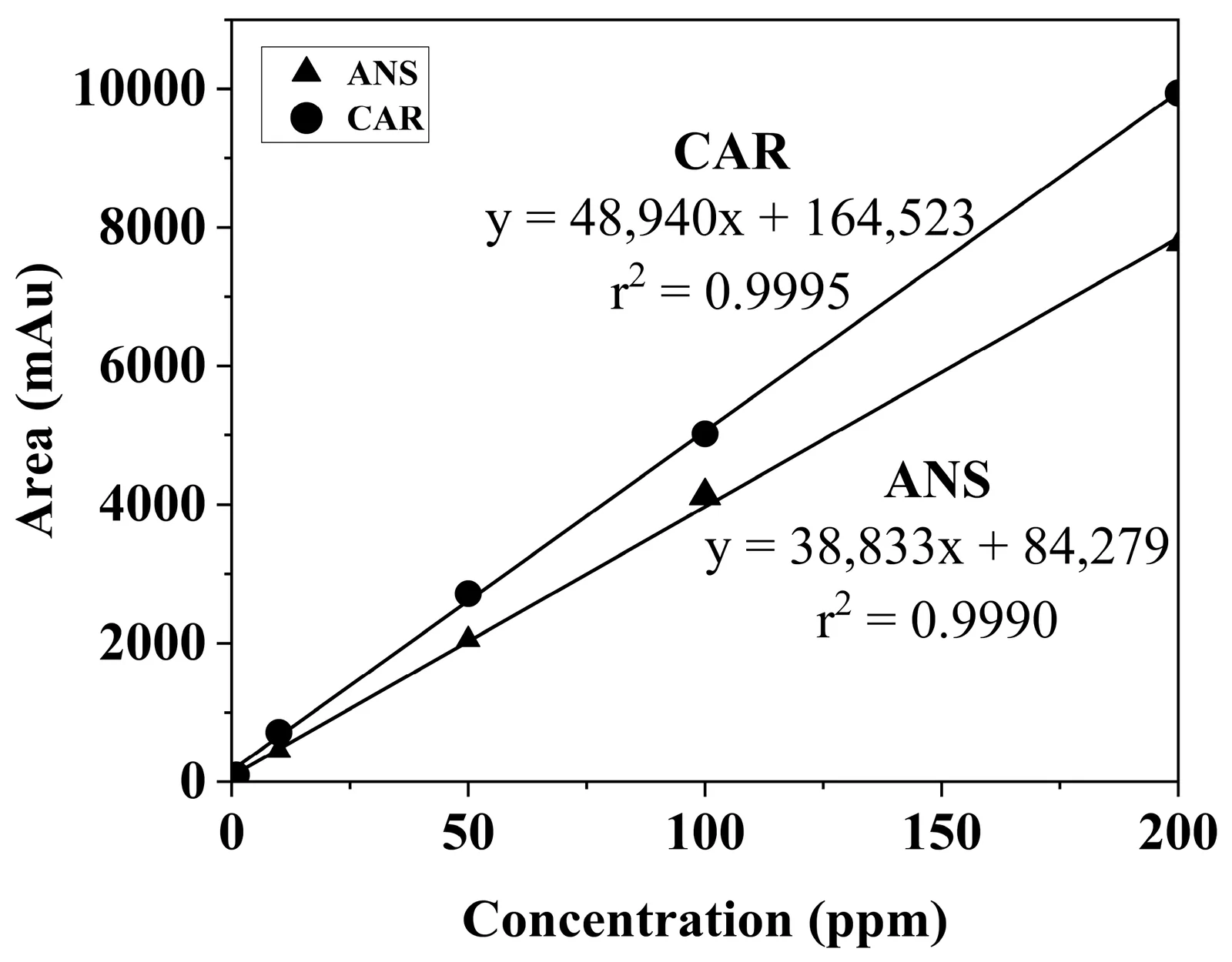
Calibration curve for anserine and carnosine.

To confirm the quantitative reliability of the developed HPLC-DAD method, a comprehensive analytical validation was performed, focusing on linearity, sensitivity, accuracy, and repeatability. Stock solutions of standard carnosine and anserine (1000 µg mL^−1^) were prepared in a 50 mM phosphate buffer solution (pH 5.0). Working standards were subsequently generated via serial dilution of the stock solutions to encompass a concentration range of 1–200 µg mL^−1^. Each calibration point was analyzed in triplicate. Under the chromatographic conditions, both analytes demonstrated excellent linearity across the entire concentration range, yielding correlation coefficients (r^2^) ≥ 0.9990 (Table 1 and Figure 2). The limits of detection (LOD) and quantification (LOQ) were calculated based on the standard deviation of the response (σ) and the slope of the calibration curve (S) using the equations:

$$\mathrm{LOD}\;=\;\frac{3.3\sigma }{S}$$

$$\mathrm{LOD}=\frac{10\sigma }{S}$$

As summarized in Table 2, the calculated LOD and LOQ values range from 0.0093 to 0.0139 mg g^−1^ and 0.0283 to 0.0420 mg g^−1^, respectively. Compared with the previously reported results, our LOD and LOQ results are in a similar range to those of HILIC, UHPLC–QqQ–MS/MS, and HPLC-MS [21,28,29]. Additionally, the HPLC-DAD extraction method employed 8% perchloric acid solution, with 4-imidazoleacrylic acid added as an internal standard [36], while this study employes a less concentrated acidic extraction condition and does not require the adding of an internal standard for the simultaneous determination of carnosine and anserine. These low detection limits underscore the high sensitivity of the developed method, confirming its suitability for the trace-level determination and routine profiling of these histidine-containing dipeptides within complex meat matrices.

#### 3.2.2. Accuracy and Repeatability

Method accuracy and repeatability were comprehensively evaluated by conducting spike–recovery experiments using chicken breast samples fortified at three nominal spiking levels (10, 50, and 200 μg mL^−1^) and are summarized in Table 3. In Table 3, the mean recoveries for both dipeptides ranged from 96.64 to 102.68%. These high recovery rates demonstrate the excellent accuracy of the method and confirm that the optimized sample preparation protocol effectively minimizes matrix-induced interference.

The associated relative standard deviation (RSD) values were consistently ≤14.18%. Although the RSD for anserine at the lowest fortification level (10 μg mL^−1^) was slightly elevated, this trend is entirely consistent with the increased analytical variability typically observed at concentrations approaching the LOQ in complex biological matrices. Crucially, even at this lowest spiking level, the precision parameters remained well within the acceptable limits defined by the Association of Official Analytical Chemists (AOAC) guidelines for trace-level analysis in food matrices [37]. Collectively, these validation parameters confirm that the proposed workflow is sufficiently robust, precise, and reliable for the routine quantification of these bioactive dipeptides across the chicken muscle matrix.

### 3.3. Quantification of Carnosine and Anserine in Diverse Meat Matrices

**Figure 3:**
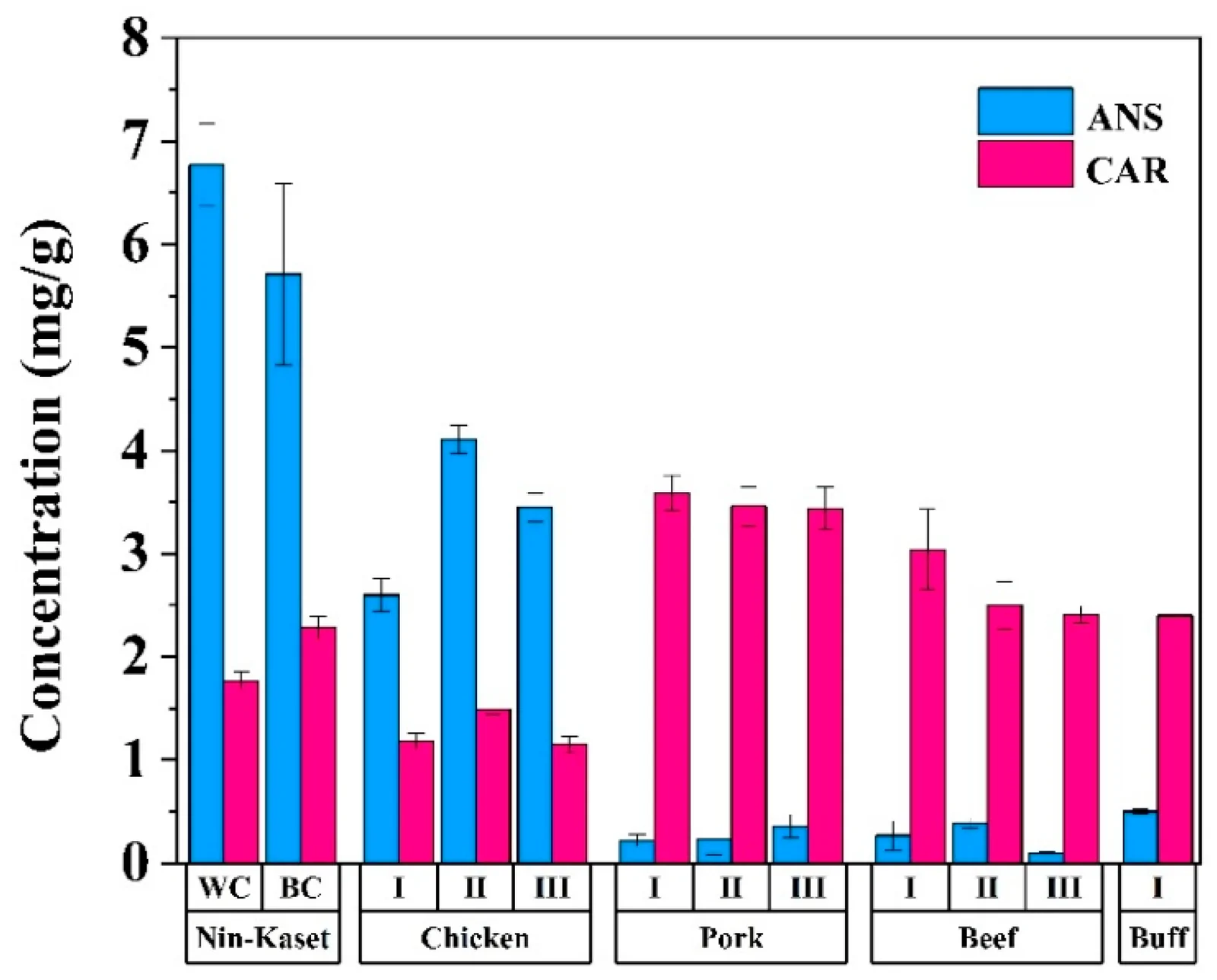
Anserine and carnosine in meat samples.

The developed extraction protocol and validated HPLC-DAD method were successfully applied to quantify the endogenous carnosine and anserine contents across a diverse suite of commercial and native meat matrices. The sample matrix included indigenous Nin-Kaset black-boned chicken, broiler, pork, beef, and buffalo meat (summarized in Table 4 and illustrated in Figure 3).

The distribution profiles of the two dipeptides exhibited distinct, species-specific patterns. In poultry samples, anserine was identified as the predominant dipeptide, ranging from 2.60 to 6.77 mg g^−1^, whereas carnosine spanned a lower range of 1.15–2.28 mg g^−1^. Conversely, red meat samples (pork, beef, and buffalo meat) exhibited substantially lower anserine levels (0.10–0.50 mg g^−1^) but were characterized by markedly higher carnosine contents (2.40–3.59 mg g^−1^).

**Figure 4:**
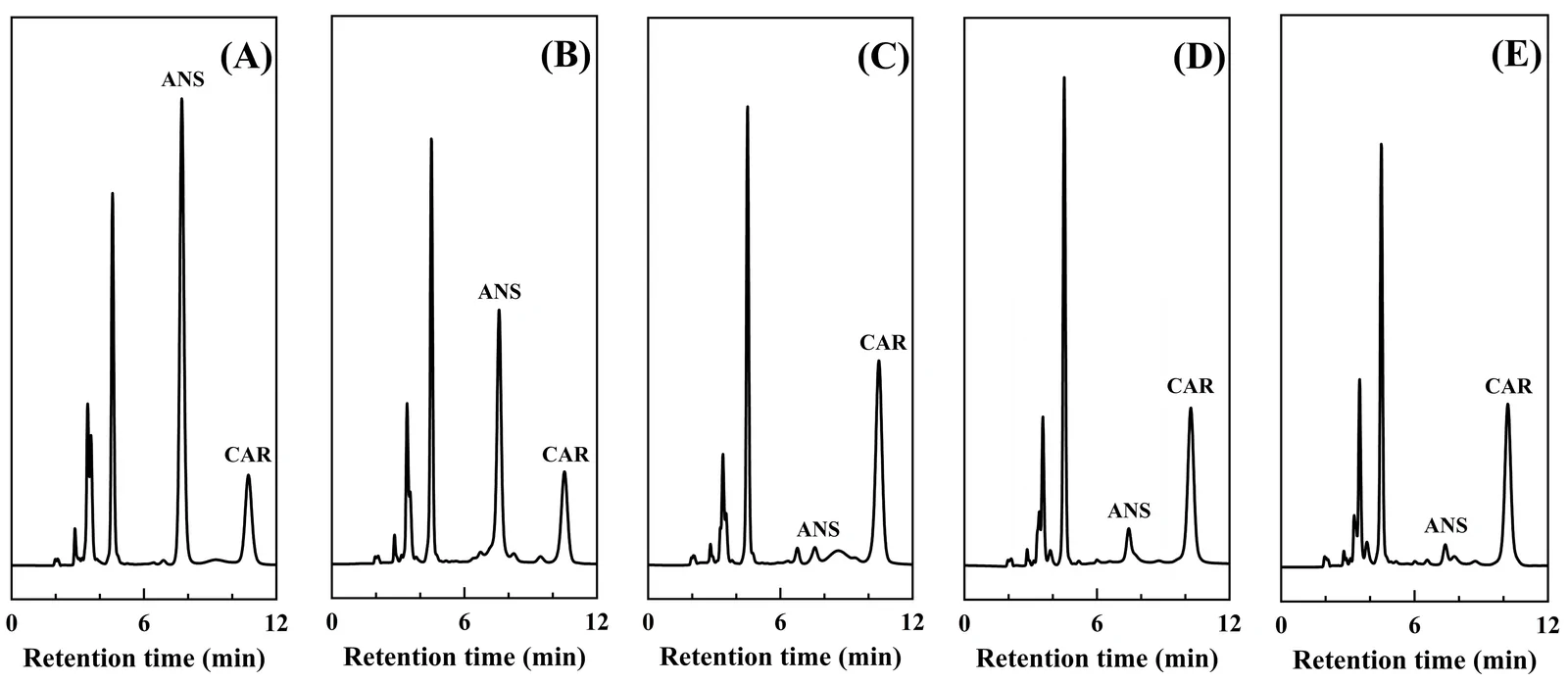
Typical HPLC-DAD chromatograms illustrate the separation of carnosine and anserine in various meat matrices. (**A**) Nin-Kaset black-boned chicken, (**B**) broiler, (**C**) pork, (**D**) beef, and (**E**) buffalo meat.

The inversion of the dipeptide profile, defined by the anserine dominance in poultry and carnosine dominance in red meat, was statistically significant (*p* < 0.001) and aligns seamlessly with the established literature [10,27]. It is worth noting that sample preparation plays a critical role in mitigating matrix effects caused by the varying lipid and protein contents in white and red meat. During this study, the lipids were extracted using 0.01 M HCl, and protein precipitation was performed using CH3CN, confirming the peak purity chromatograms in Figure 4. Notably, the validation method was performed using the broiler breast. To visually confirm these quantitative trends and demonstrate the high chromatographic resolution of the developed method when handling complex, matrix-dense samples, the typical chromatograms for the Nin-Kaset black-boned chicken, broiler, pork, beef, and buffalo meat samples are presented in Figure 4, highlighting excellent peak separation with minimal baseline noise. This highlights a simple and applicable method for the routine analysis of both compounds in meat samples.

### 3.4. Comparison of Thai Native Chicken (Nin-Kaset) vs. Commercial Breeds

Notably, the white-feather Thai native chicken breast exhibited significantly higher anserine concentrations compared to the commercial broiler (*p* < 0.001). Meanwhile, the highest carnosine concentrations were found in pork (*p* < 0.001), while the black-feather Thai native chicken breast maintained an elevated carnosine profile comparable to that of beef. This metabolic divergence in dipeptide distribution across species is biochemically governed by the activity of carnosine N-methyltransferase, an enzyme encoded by the histamine N-methyltransferase-like (HNMT-like) gene. Within avian species, this enzyme catalyzes the methylation of carnosine to form anserine. The specific HNMT-like gene is evolutionarily absent from mammalian genomes [38]. Consequently, anserine is the dominant histidine-containing dipeptide in poultry, whereas carnosine predominates in red meats. Remarkably, the indigenous Nin-Kaset chicken demonstrated a distinct nutritional advantage, exhibiting anserine and carnosine concentrations approximately 1.5-fold and 2.0-fold higher, respectively, than those of broiler. These elevated dipeptide concentrations are likely modulated by breed-specific physiological characteristics. These include variations in skeletal muscle fiber composition, enhanced oxidative metabolism [24,39], higher antioxidant demand [24,25], and slower growth-related metabolic maturation [40,41]. Collectively, these results highlight the strong potential of Nin-Kaset chicken as a functional food source naturally enriched with bioactive dipeptides. Furthermore, the analytical framework validated in this study offers a highly practical, repeatable, and routine methodology for the determination of carnosine and anserine across diverse food and meat matrices.

## 4. Conclusions

This study demonstrates a rapid, simple, and derivatization-free HPLC–DAD methodology for the simultaneous quantification of carnosine and anserine across diverse meat matrices. We successfully simplified the extraction method using a low concentration of acidic solution and stabilized the carnosine and anserine structures by keeping them in adjusted phosphate solution using 0.01 M potassium hydroxide solution. Strategic pK_a_ adjustment at pH 5.0 with a 3:1 CH_3_CN:adjusted phosphate solution ratio proved to be crucial in controlling the protonation states of both dipeptides. This pH-controlled stabilization effectively eliminated chemical speciation heterogeneity, thereby ensuring superior chromatographic resolution and excellent peak symmetry at ambient temperature without the requirement of pre-column derivatization. Rigorous analytical validation confirmed the reliability and suitability of this method for routine analysis, demonstrating excellent linearity (r^2^ ≥ 0.9990), satisfactory sensitivity (LOD: 0.0093–0.0139 mg g^−1^; LOQ: 0.0283–0.0420 mg g^−1^), acceptable recovery (96.64–102.68%), and robust repeatability (RSD ≤ 14.18%). Application of the developed method across various species successfully mapped their distinct, species-specific dipeptide profiles, reinforcing that anserine predominates in poultry (2.60–6.77 mg g^−1^), while carnosine is the primary dipeptide in red meats (2.40–3.59 mg g^−1^). Remarkably, the indigenous Nin-Kaset black-boned chicken was identified as a premium functional food source, exhibiting approximately 2.0-fold higher carnosine and 1.5-fold higher anserine levels than commercial broiler. Method validation in this work focused on poultry meat as a proof of concept for the muscle matrix analysis. The sample preparation step successfully mitigates lipid and protein interferences. The extensions of this work aim to expand to full multi-laboratory for validation across broader red meat categories such as pork and beef in future work. Beyond establishing Nin-Kaset chicken as a superior source of bioactive dipeptides, this study provides a sustainable and efficient tool for routine food profiling. This approach aligns with a more streamlined and practical alternative to derivatization-heavy methods and offers a practical solution for large-scale nutritional evaluation in the food industry.

## Figures and Tables

**Table 1:** Linearity parameters, limit of detection (LOD), and limit of quantification (LOQ) for anserine and carnosine.

Compounds	Linear Range (µg mL^−1^)	Regression Equation	r^2^	LOD (mg g^−1^)	LOQ (mg g^−1^)
Anserine	1–200	y = 38,833x + 84,279	0.9990	0.0139	0.0420
Carnosine	1–200	y = 48,940x + 164,523	0.9995	0.0093	0.0283

**Table 2:** Comparison of performance of the developed method with recently reported values for quantification of anserine and carnosine.

Analyte	Techniques	LOD (mg g^−1^)	LOQ (mg g^−1^)	References
Carnosine anserine	HILIC	0.00564 0.00823	-	[21]
Carnosine anserine	UHPLC–QqQ–MS/MS	0.00012 0.00177	0.00036 0.00538	[29]
Carnosine anserine	HPLC-MS	-	0.00234 0.00116	[28]
Carnosine anserine	HPLC-DAD	0.0210 0.0195	0.0700 0.0645	[36]
Carnosine anserine	HPLC-DAD	0.0093 0.0139	0.0283 0.0420	This work

**Table 3:** Recovery and repeatability of carnosine and anserine in spiked chicken breast samples (*n* = 5).

Compounds	Spiked Concentration (µg g^−1^) ^1^	Theoretical Concentration (µg g^−1^) ^2^	Found Concentration (µg g^−1^) ^3^	Recovery (%) ^4^	RSD (%) ^5^
Anserine	9.84	27.11	27.38	102.68	14.18
	51.51	70.19	70.66	100.90	4.44
	168.82	182.67	184.09	100.84	1.68
Carnosine	10.36	16.47	16.12	96.64	7.92
	51.27	56.73	57.00	100.53	1.50
	138.03	140.05	139.80	99.82	0.70

^1^ Spiked concentration = (Peak area − Intercept)/Slope. ^2^ Theoretical concentration = Unspiked sample + Spiked concentration. ^3^ Found concentration = Spiked sample. ^4^ Recovery (%) = (Found − Unspiked sample)/Spiked sample × 100%. ^5^ RSD = (SD/mean) × 100%.

**Table 4:** Anserine and carnosine in meat samples (*n* = 5).

Meat Samples	Average ± SD (mg g^−1^)	
	Anserine	Carnosine
White-feather Thai native chicken breast	6.77 ± 0.40 ^a^	1.77 ± 0.09 ^d^
Black-feather Thai native chicken breast	5.71 ± 0.88 ^b^	2.28 ± 0.11 ^c^
Broiler breast I *	2.60 ± 0.16 ^e^	1.18 ± 0.08 ^f^
Broiler breast II *	4.11 ± 0.14 ^c^	1.49 ± 0.05 ^e^
Broiler breast III *	3.45 ± 0.14 ^d^	1.15 ± 0.08 ^f^
Pork sirloin I *	0.23 ± 0.06 ^f^	3.59 ± 0.19 ^a^
Pork sirloin II *	0.22 ± 0.15 ^f^	3.46 ± 0.17 ^a^
Pork sirloin III *	0.36 ± 0.11 ^f^	3.44 ± 0.21 ^a^
Ribeye beef I *	0.27 ± 0.14 ^f^	3.04 ± 0.39 ^b^
Ribeye beef II *	0.39 ± 0.05 ^f^	2.50 ± 0.23 ^c^
Ribeye buff I *	0.50 ± 0.02 ^f^	2.40 ± 0.17 ^c^

* I, II, and III represented the purchase samples from the supermarket with the various suppliers. Different superscript letters (a–f) within the same column indicate significant differences (*p* < 0.001) according to Duncan’s multiple-range test.

## Data Availability

The datasets generated and analyzed during the current study are available within the paper. Any additional information is available from the corresponding author upon request.

## Supplementary Materials

The following supporting information can be downloaded at: https://www.mdpi.com/article/10.3390/mps9050131/s1, Figure S1: Typical HPLC-DAD chromatograms illustrate the effect of different solvents on the separation of carnosine and anserine mix standards (300 µg mL-1): (A) mixture of acetonitrile and phosphate buffer solution (CH_3_CN:buffer, 3:1 *v*/*v*), (B) phosphate buffer solution, and (C) ultrapure water.
