# Supplementary material for: Quantification of Carnosine and Anserine in Diverse Meat Matrices via Optimized Extraction and Validated HPLC-DAD
Source: Methods Protoc. 2026 Sep 4;9(5):131. doi: 10.3390/mps9050131 (PMC13600070; doi:10.3390/mps9050131)
Supplement: Supplementary file 1 [file mps-09-00131-s001.zip › mps-4482320-supplementary.pdf]

Data availability

Optimized of the simplified extraction protocol

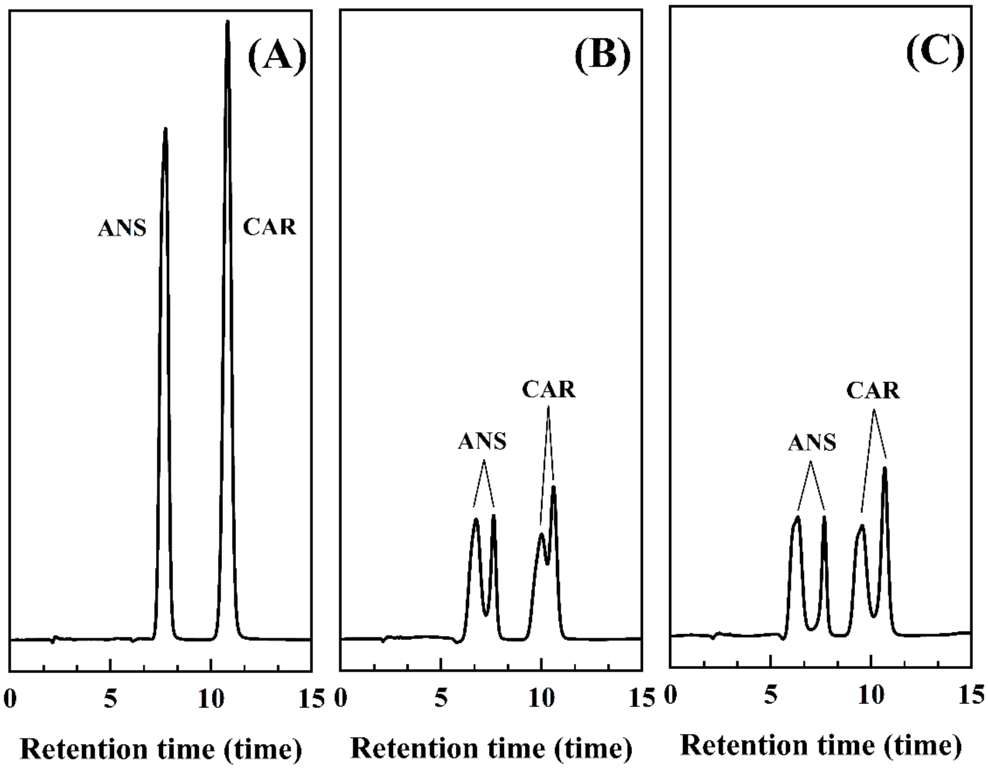

Figure S1. Typical HPLC-DAD chromatograms illustrate the effect of different solvents on the separation of carnosine and anserine mix standards (300 μg mL^{-1}): (**A**) mixture of acetonitrile and phosphate buffer solution (CH_3CN:buffer, 3:1 v/v), (**B**) phosphate buffer solution, and (**C**) ultrapure water.

To systematically evaluate the impact of the solvent on the separation of carnosine and anserine mix standard (300 μg mL^{-1}) were prepared in three different solvent systems. As illustrated in Figure S1, using ultrapure water (Figure S1c) severely compromised the chromatographic resolution, resulting in insufficient peak separation and significant peak broadening for both standards. Conversely, the phosphate buffer solution (Figure S1b) yielded higher resolution than ultrapure water, which can be attributed to its stabilization of the pH around the second dissociation constant (pK_{a2}) of both compounds. However, these results indicated that controlling the pH is insufficient for achieving complete chromatographic separation.

Consequently, the inclusion of acetonitrile (CH_3CN) was found to be indispensable for achieving baseline separation and optimal peak shapes. As shown in Figure S1a, incorporating CH_3CN into the phosphate buffer solution (CH_3CN:buffer, 3:1 v/v) as a buffer modifier dramatically enhanced the separation efficiency[1]. This improvement is attributed to the role of organic modifier in stabilizing the ionization states of carnosine and anserine, thereby preventing doublet peak of multiple ionic forms.

The datasets generated and analyzed during the current study are available within the paper. Any additional information is available from the corresponding author upon request.

(1) Bean, S. R.; Lookhart, G. L.; Bietz, J. A. Acetonitrile as a Buffer Additive for Free Zone Capillary Electrophoresis Separation and Characterization of Maize (Zea mays L.) and Sorghum (Sorghum bicolor L. Moench) Storage Proteins. *J. Agric. Food. Chem.* **2000**, *48* (2), 318-327. DOI: https://doi.org/10.1021/jf990786o.
